# Clinical features of Parkinson’s disease with and without rapid eye movement sleep behavior disorder

**DOI:** 10.1186/s40035-017-0105-5

**Published:** 2017-12-21

**Authors:** Ye Liu, Xiao-Ying Zhu, Xiao-Jin Zhang, Sheng-Han Kuo, William G. Ondo, Yun-Cheng Wu

**Affiliations:** 1 Department of Neurology, Shanghai General Hospital, Shanghai Jiao Tong University School of Medicine, No.100, Haining Road, Shanghai, 200080 People’s Republic of China; 2 Department of Neurology, College of Physicians and Surgeons, Columbia University, New York, USA; 3 Methodist Neurological Institute, Houston, TX USA

**Keywords:** Rapid eye movement sleep behavior disorder, Parkinson’s disease, Depression, Cognitive decline, Orthostatic hypotension, Motor deficits

## Abstract

**Background:**

Rapid eye movement sleep behavior disorder (RBD) and Parkinson’s disease (PD) are two distinct clinical diseases but they share some common pathological and anatomical characteristics. This study aims to confirm the clinical features of RBD in Chinese PD patients.

**Methods:**

One hundred fifty PD patients were enrolled from the Parkinson`s disease and Movement Disorders Center in  Department of Neurology, Shanghai General Hospital from January 2013 to August 2014. This study examined PD patients with or without RBD as determined by the REM Sleep Behavior Disorder Screening Questionnaire (RBDSQ), assessed motor subtype by Unified PD Rating Scale (UPDRS) III at “on” state, and compared the sub-scale scores representing tremor, rigidity, appendicular and axial. Investigators also assessed the Hamilton Anxiety Scale (HAMA), Hamilton Depression Scale (HAMD), Mini-Mental State Examination (MMSE), Clinical Dementia Rating (CDR), and Parkinson’s disease Sleep Scale (PDSS).

**Results:**

One hundred fourty one PD patients entered the final study. 30 (21.28%) PD patients had probable RBD (pRBD) diagnosed with a RBDSQ score of 6 or above. There were no significant differences for age, including age of PD onset and PD duration, gender, smoking status, alcohol or coffee use, presence of anosmia or freezing, UPDRS III, and H-Y stages between the pRBD^+^ and pRBD^−^ groups. pRBD^+^ group had lower MMSE scores, higher PDSS scores, and pRBD^+^ PD patients had more prominent proportion in anxiety, depression, constipation, hallucination and a greater prevalence of orthostatic hypotension.

**Conclusion:**

pRBD^+^ PD patients exhibited greater changes in non-motor symptoms. However, there was no increase in motor deficits.

## Background

Rapid eye movement sleep (REM) is characterized by decreased or absent muscle tone (atonia), desynchronization of the electroencephalogram, with the presence of saw tooth waves, and autonomic instability. Rapid eye movement sleep behavior disorder (RBD) is a form of parasomnia during which patients develop limb or body movements, which correlate with dream enactment behavior. The abnormal physiology of RBD is loss of muscle atonia (paralysis) during otherwise intact REM sleep [1].

The standard RBD diagnostic criteria are based on the 2nd edition of the International Classification of Sleep Disorders (ICSD) [2], and polysomnology (PSG) is necessary for a definitive diagnosis. Nomura et al., determined that RBD rapid screening questionnaire (RBDSQ), which is completed by the patient, had a sensitivity of 84.2% and specificity of 96.2% to diagnose RBD when compared with standard RBD diagnostic criteria using PSG in PD at a cut off of 6 points (total score of RBDSQ is 13, and a score of 5 is the cut-off point for healthy individuals) [3]. Chahine et al. investigated the use of the RBDSQ plus Mayo Sleep questionnaire 1 (MSQ1) compared with PSG in PD patients. They found sensitivity was highest when the questionnaires were used in combination while specificity was highest for the RBDSQ used alone at a cut-off point of 7 [4]. Shen SS used the RBDSQ to diagnose RBD in Chinese patients, compared with PSG, and found a cutoff points at 6 had the best specificity and sensitivity [5].

RBD has a close relationship with neurodegenerative diseases, especially those with α-synucleinopathy pathology such as PD, dementia with Lewy bodies (DLB) and multiple system atrophy (MSA) [1, 6]. Recently, studies have indicated that PD patients with RBD might have some specific clinical features more commonly than those without RBD. Although data is mixed, PD patients with RBD have been reported to have worse decision-making [7], cognitive impairment [6], freezing, falls and rigidity [8, 9]. They also have a higher prevalence of orthostatic hypotension (OH) [8] and visual hallucinations (VH) [10]. However, there are no detailed data in PD patients with RBD in China. In this paper, we investigated the clinical features of PD patients with RBD in a tertiary referral center in China. The present study focused on the characteristics of motor and non-motor symptoms of PD with RBD compared to PD without RBD.

## Methods

### Patient selection

One hundred fifty PD patients were enrolled from the Parkinson’s disease and Movement Disorder Center in the Department of Neurology, Shanghai General Hospital, Shanghai Jiao Tong University, Shanghai, China from January 2013 to August 2014. The diagnosis of PD was made by two movement disorder specialists according to the UK Parkinson’s Disease Society (UKPDS) Brain Bank Criteria. Patients with severe dementia (CDR ≥ 2), or other central nervous system disorders were excluded from this study. The study was approved by the Institution’s Ethics Committee and all recruited patients consented to participate in the study.

### Patient evaluation

Of 150 patients, 5 patients were excluded because of probable DLB, 3 patients had probable MSA, and 1 patient had vascular Parkinsonism. A total of 141 patients were enrolled in the study. Patient evaluation was performed by movement disorder specialists. Parkinsonism staging was evaluated according to the Hoehn & Yahr staging scale. Part III of the Unified PD Rating Scale (UPDRS III) was performed during the “on” state. Motor subtype was analyzed by predominance of tremor or rigidity (UPDRS sub-scores) and predominance of limb or axial features (UPDRS sub-scores) [9], Hamilton Anxiety Scale (HAMA; cut-off point ≥8), Hamilton depression Scale (HAMD; cut-off point ≥8) and Mini-Mental State Examination (MMSE) were used to evaluate the patient’s mood and cognitive state. The patients whose MMSE score was lower than 17 (illiteracy) or lower than 20 (primary school level) or 24 (higher than middle school cultural level) [11] were considered to have dementia and were evaluated using the Clinical Dementia Rating (CDR). Patients whose CDR was higher than 2 were excluded from the study. Investigators used the REM Sleep Behavior Disorder Screening Questionnaire(RBDSQ)to detect clinical probable RBD (pRBD). We set the RBDSQ cut-off point at a score of 6 according to the highest sensitivity and specificity determined by a previous study [5]. The Parkinson’s disease Sleep Scale (PDSS) was used to evaluate patient’s sleep quality. Orthostatic hypotension (OH) was screened using a simple question:“Do you feel dizziness or weakness when you stand up?” If the answer was “yes”, a blood pressure test from the supine to standing position was checked, a fall in systolic blood pressure of ≥20 mmHg, or in diastolic blood pressure of ≥10 mmHg, was diagnosed as OH. Other items including smoking, alcohol and coffee consumption, hyposmia or anosmia, constipation were also documented by question. Patients continued with their prescribed treatment regimen, they used anti-Parkinson drugs, anti-hypnotics or anti-depressants as necessary.

### Statistical analysis

The data were analyzed using the Statistical Package for Social Sciences (SPSS) 19(IBM Co., USA). The data is presented as mean, counts and percentages, and the adjusted difference in means. Analysis of descriptive variables was performed using two-tailed t tests. Mann-Whitney U tests and X^2^ tests were used where appropriate. A P-P plot was used to test normal distribution. A *p* value <0.05 was considered to be significant.

## Results

### Demographics

Among 141 patients, 74 were male (52.48%): 18 male (60%) in pRBD^+^ group, and 56 male (50.45%) in pRBD^−^ group (*p* = 0.655). Thirty patients (21.28%) were diagnosed with probable RBD (pRBD) based on a RBD screening questionnaire score ≥ 6. If the cut-off score was set at 7 or 5, the incidence of RBD was 17.02% and 26.24% respectively, with little difference in the clinical features (Table 3). The mean age in the pRBD^+^ group was 68.33 ± 8.76 versus 69.32 ± 9.75 years in the pRBD^−^ group (*p* = 0.618). Mean PD duration years is 4.13 ± 4.216 in pRBD^+^ and 4.65 ± 3.570 in pRBD^−^ group (*p* = 0.5). Smoking, alcohol and coffee consumption were infrequent in both groups (NS). There was no difference between the numbers of patients who took levodopa or dopamine agonists, and the levodopa equivalent dosage was similar in both groups. However, the pRBD^+^ group had greater antidepressant and anti-hypnotic use (Table 1).

**Table 1:** Epidemiological characteristic of PD patients

	pRBD+	pRBD-	*p*
Patients number (*n*)	30	111	
Age (yr)	68.33 ± 8.76	69.32 ± 9.75	0.618
PD duration (yr)	4.13 ± 4.22	4.65 ± 3.57	0.500
Gender, male, *n* (%)	18 (60.00)	56 (50.45)	0.655
Smoking, *n* (%)	4 (13.3)	11 (9.91)	0.525
Alcohol, *n* (%)	0	9 (8.11)	0.204
Coffee, *n* (%)	1 (3.33)	5 (4.50)	1.0
Levodopa dose equivalent (mg/day)	353.53 ± 236.10	339.10 ± 272.08	0.531
Levodopa dose (mg/day)	325.00 ± 205.84	285.15 ± 250.09	0.221
Levodopa years	2.59 ± 3.08	3.03 ± 3.31	0.577
Dopa agonist dose (mg/day)	38.23 ± 51.01	33.19 ± 45.12	0.931
Dopa agonist years	0.91 ± 1.52	4.41 ± 29.21	0.479
TCA(Deanxit), *n* (%)	1 (0.033)	1 (0.009)	>0.90
Trihexyphenidyl, *n* (%)	4 (0.133)	18 (0.162)	>0.90
SSRI, *n* (%)	2 (0.067)	3 (0.027)	>0.75
BNZ, *n* (%)	6 (0.2)	3 (0.027)	<0.005

*SSRI* Selective Serotonin Reuptake Inhibitor

*BNZ* Benzodiazepines, n: number

The age, mean PD duration years, smoking, alcohol and coffee consumption rates, levodopa had no difference between two groups. However, the pRBD^+^ group had greater antidepressant and antihypnotic use

### Non-motor symptoms

pRBD^+^ PD patients had significantly higher rates of anxiety (60% vs 22.52%, <0.001) and depression (63.33% vs 24.32%,*p* < 0.001) (Table 2), and a higher mean PDSS score (17.50 ± 9.60 vs 11.70 ± 8.55, p < 0.001) (Table 2). The pRBD^+^ group had lower mean MMSE score (25.30 ± 3.91 vs. 26.75 ± 3.97, *p* = 0.017) (Table 3). When educational status was taken into account, a diagnosis of dementia was not significantly difference between two groups (16.67% vs 11.71%, *p* = 0.538) (Table 2). Constipation and OH were more prominent in the pRBD^+^ group (Table 2).

**Table 2:** Clinical characteristics of PD patients

	pRBD+	pRBD-	*p*
Hoehn & Yahr stage	2.40 ± 0.90	2.26 ± 0.90	0.299
UPDRS III(total)	26.93 ± 14.62	23.68 ± 15.93	0.174
Bradykinesia (23–26)	8.87 ± 7.05	7.40 ± 6.11	0.292
Freezing, *n* (%)	8 (26.7)	25 (22.52)	0.750
LcompareA	5.67 ± 4.22	6.17 ± 4.61	0.734
RcompareT	3.01 ± 4.06	1.79 ± 2.08	0.320
PDSS	17.50 ± 9.60	11.70 ± 8.55	<0.001
MMSE	25.30 ± 3.91	26.75 ± 3.97	0.017
HAMA	7.53 ± 5.51	4.53 ± 6.38	0.002
HAMD	10.83 ± 8.60	5.39 ± 7.12	<0.001
Hallucination, *n* (%)	6 (20.00)	7 (6.30)	0.032
Dementia, *n* (%)	5(16.67)	13(11.71)	0.538
Anxiety, *n* (%)	18 (60.00)	25 (22.52)	<0.001
Depression, *n* (%)	19 (63.33)	27 (24.32)	<0.001
Hyposmia, %	46.67	35.16	0.247
Constipation, %	80.00	57.66	0.033
Orthostatic hypotension, *n* (%)	7 (23.33)	7 (6.30)	0.012

LcompareA (UPDRS III): UPDRS score in limb compare with axial; RcompareT (UPDRS III): UPDRS score in rigidity compare with tremor; *n* Number

The pRBD^+^ PD patients had significantly higher rates of anxiety, depression and visual hallucinations rates. And they had a higher mean PDSS score. Dementia was not significantly difference between two groups. Constipation and OH were more prominent in the pRBD^+^ group

**Table 3:** The clinical difference of RBD^+^ vs RBD^−^ patients while RBDSQ cutoff at 7, 6 and 5 in present study

	7 score	6 score	5 score
RBD incidence, *n* (%)	24 (17.02)	30 (21.28)	37 (26.24)
Age	–	–	–
PD duration	–	–	–
Gender	–	–	–
Dopamine use	–	–	–
Hypnosmia	–	–	–
Freezing	–	–	–
Hallucination	+	+	–
Constipation	+	+	+
HAMA	+	+	+
HAMD	+	+	+
PDSS	+	+	+
MMSE	+	+	+
Orthostatic hypotension	+	+	+
UPDRS III (total)	–	–	–
Bradykinesia (23–26)	–	–	–
LcompareA	–	–	–
RcompareT	–	–	–

If the cut off score was set at 7, 6, or 5, the clinical features had little difference. If the cut off score was set at 5 score, the hallucination rates would have no difference in two groups instead. The other features were same in the three condition

There was no difference in the incidence of anosmia in pRBD^+^ vs pRBD^−^ group (46.67% vs 35.16%, *p* = 0.247) (Table 2). From PDSS item 7, we found that there were 13 patients who had visual hallucinations, 20% (6) in pRBD^+^ group and 6.3% (7) in pRBD^−^ group (*p* = 0.032) (Table 2).

### Motor symptoms

The UPDRS III score was similar in the pRBD^+^ and pRBD^−^ groups (26.93 ± 14.62 vs 23.68 ± 15.93, *p* = 0.174). Mean H-Y stage was also similar between the two groups (2.40 ± 0.90 vs 2.26 ± 0.90, *p* = 0.299). When limb scores in UPDRS III was compared with axial scores, the ratio showed no difference between pRBD^+^ and pRBD^−^ groups (5.67 ± 4.22 vs 6.17 ± 4.61, *p* = 0.734). When rigidity scores in UPDRS III were compared with resting tremor score, the ratio was also not significantly different (3.01 ± 4.06 vs 1.79 ± 2.08, *p* = 0.32). Overall there was no difference between pRBD^+^ group and pRBD^−^ group for motor severity and motor subtype (Table 2). Freezing was also not different between the two groups (26.7% vs 22.52%, *p* = 0.75).

## Discussion

Probable RBD is common in early PD and predicts future cognitive decline, particularly in attention and memory domains [12]. The pedunculopontine nucleus (PPN) and locus cerulean (LC)/ dorsal subcoeruleus (subCD) are compromised in both PD and RBD [13–15]. Autopsy studies show that the loss of cholinergic neurons of the PPN in PD has a significant negative correlation with the modified Hoehn and Yahr stage [16], and contribute to freezing and falls [16, 17]. Dysfunction of the PPN relates to visual hallucination (VH) [15]. A resting-state functional connectivity MRI (rs-fcMRI) study in RBD patient showed reduced connection between lateral geniculate nuclei LGN and visual association cortex [18]. PD patients with probable RBD showed smaller volumes than patients without RBD and than healthy controls in the pontomesencephalic tegmentum where cholinergic, GABAergic and glutamatergic neurons are located. It is additionally associated with more widespread atrophy in other subcortical and cortical regions [19]. The basal ganglia activity is changed across the sleep-wake cycle in RBD [20]. The appearance of RBD in PD may be related to regional gray matter changes in the left posterior cingulate and hippocampus but not localized to the brain stem [21].

Our study had compared motor and non-motor symptoms in PD patients with and without RBD. The results were similar if we used a RBDSQ cut off point at 6 or 7 (Table 3). Overall motor symptoms and signs were similar but we found significant difference between the two groups in many aspects of non-motor symptoms, including MMSE performance, visual hallucinations, depression, anxiety, orthostatic hypotension and constipation. Except for constipation, these results are consistent with most previous studies (detailed in Table 4) [7, 22–24]. Our findings are consistent with some studies that there are no difference between the motor symptoms [13, 23], however, some previous studies indicated that pRBD+ patients showed much more worse in the gait, balance or increased dyskinesia (Table 4) [8, 9, 22, 25].

**Table 4:** Clinical features of PD with RBD patients

	Present study	Sixel-Doring 2014	Yoritaka 2009	Vibha 2011	Romenets 2012	Sommerauer 2014	Rolinski 2014
Patients(*n*)	141	158	150	134	98	59	475
RCP	6	no	^b^	^b^	no	no	5
RBD (%)	21.28	51.27	–	19.4	–	–	47.2
Male pro	–	–	+	–	+	–	+
older	–	–	+	–	+	–	–
MMSE	+	–	–	–	no	no	+
PDSS	+	no	no	+	no	no	^a^
Depression/anxiety	+	no	no	–	+	no	+
Dementia	–	no	–	no	ex	no	+
Hallucination	+	no	–	+	–	no	+
N-Tremor prominent	–	–	–	–	+	–	–
Axial/limb ratio	–	–	no	no	–	no	+
Constipation	+	no	+	no	–	no	+
Hyposmia	–	–	no	no	–	no	–
OH	+	no	–	no	+	no	+
PSG	N	Y	N	N	Y	Y	N

-: no difference, +: significant difference

Ex: dementia patient was excluded

*Male pro* Male prominent

No: no data

^a^Rolinski use Epworth Sleeping Scale instead of PDSS, difference is significant

^b^Yoritaka and Vibha use minimum clinical criteria of ICSD for RBD diagnosis instead

*N* No

*N-tremor prominent* Non-tremor prominent

*OH* Orthostatic hypotension

*Y* Yes

*RCP* RBDSQ cutoff points

Overall motor symptoms and signs were similar but we found significant difference between the two groups in many aspects of non-motor symptoms, including MMSE performance, visual hallucinations, depression, anxiety, orthostatic hypotension and constipation. Except for constipation, these results are consistent with most previous studies

In summary, this study systematically investigated the clinical features of PD patients with RBD. There are several potential weaknesses. We used the RBDSQ to detect RBD in PD patients which is easier and more readily available than PSG. The sample size is relatively small and it might have given false negatives and false positives for diagnosing RBD without PSG. We diagnosed the patient with anosmia and constipation only based on self-report and not using objective examination. Neurological image and electrophysiology will be valuable for further study.

## Conclusions

The present study demonstrated that there were no significant differences in motor deficits in pRBD^+^ PD patients, while the non-motor symptoms are prominent, such as mood, sleep, constipation, cognition and orthostatic hypotension. However, further studies and laboratory tests are needed to improve the understanding of RBD in PD.

## Abbreviations

CDR: Clinical Dementia Rating
DLB: Dementia with Lewy bodies
HAMA: Hamilton Anxiety Scale
HAMD: Hamilton Depression Scale
ICSD: International Classification of Sleep Disorders
LC: Locus cerulean
MMSE: Mini-Mental State Examination
MSA: Multiple system atrophy
MSQ1: Mayo Sleep questionnaire 1
OH: Orthostatic hypotension
PD: Parkinson’s disease
PDSS: Parkinson’s disease Sleep Scale
PPN: Pedunculopontine nucleus
pRBD: Probable RBD
PSG: Polysomnology
RBD: Rapid eye movement sleep behavior disorder
RBDSQ: REM Sleep Behavior Disorder Screening Questionnaire
subCD: Dorsal subcoeruleus
VH: Visual hallucinations
